# Pathways Community Care Coordination in Low Birth Weight Prevention

**DOI:** 10.1007/s10995-014-1554-4

**Published:** 2014-08-20

**Authors:** Sarah Redding, Elizabeth Conrey, Kyle Porter, John Paulson, Karen Hughes, Mark Redding

**Affiliations:** 1 Community Health Access Project, Columbus, OH USA; 2 State Maternal and Child Health Epidemiologist CDC Assignee, Ohio Department of Health, Columbus, OH USA; 3 Ohio State University Center for Biostatistics, Columbus, OH USA; 4 Center for Public Health Statistics and Informatics, Ohio Department of Health, Columbus, OH USA; 5 Division of Family and Community Health Services, Ohio Department of Health, Columbus, OH USA

**Keywords:** Low birth weight prevention, Community health worker, Community care coordination, Social determinants of health, Pay for performance, Home visiting

## Abstract

The evidence is limited on the effectiveness of home visiting care coordination in addressing poor birth outcome, including low birth weight (LBW). The Community Health Access Project (CHAP) utilizes community health workers (CHWs) to identify women at risk of having poor birth outcomes, connect them to health and social services, and track each identified health or social issue to a measurable completion. CHWs are trained individuals from the same highest risk communities. The CHAP Pathways Model is used to track each maternal health and social service need to resolution and CHWs are paid based upon outcomes. We evaluated the impact of the CHAP Pathways program on LBW in an urban Ohio community. Women participating in CHAP and having a live birth in 2001 through 2004 constituted the intervention group. Using birth certificate records, each CHAP birth was matched through propensity score to a control birth from the same census tract and year. Logistic regression was used to examine the association of CHAP participation with LBW while controlling for risk factors for LBW. We identified 115 CHAP clients and 115 control births. Among the intervention group there were seven LBW births (6.1 %) compared with 15 (13.0 %) among non-CHAP clients. The adjusted odds ratio for LBW was 0.35 (95 % confidence interval, 0.12–0.96) among CHAP clients. This study provides evidence that structured community care coordination coupled with tracking and payment for outcomes may reduce LBW birth among high-risk women.

## Introduction

Infant mortality rates are used as an indicator for the health of a community. To prevent infant deaths, mothers need to be healthy, live in a safe environment, and have access to quality care. Reducing low birth weight (LBW) and premature births has been identified as a key strategy to decrease infant mortality [1]. While infant mortality rates in the US have improved over the past decades, they have been stagnant in Ohio. In fact, Ohio ranked second worst for black infant mortality among all states, and fourth worst for overall infant mortality in 2010 [2, 3]. Nationally, despite overall improvements, the 2011 Centers for Disease Control (CDC) Health Disparities and Inequalities Report showed that large disparities in infant mortality rates persist [4].

Strategies that incorporate the community and directly reach out to women at greatest risk for poor birth outcomes may help communities move towards health equality. Home visiting services are one strategy used to improve birth outcomes and have received increased attention and focus on providing evidence-based services to vulnerable children and families through the Affordable Care Act and the Maternal, Infant, and Early Childhood Home Visiting (MIECHV) program [5]. Although home visiting has been shown to be effective in impacting parent behaviors, child cognitive outcomes and maternal life course, the impact on birth outcomes is not as clearly evident [6, 7].

The Community Health Access Project (CHAP) is a nonprofit, community based organization that has been providing care coordination services in Richland County, Ohio since 1999. CHAP utilizes community health workers (CHWs) to identify women at risk of having poor birth outcomes, connect them to health and social services, and track each identified issue to a measurable completion. CHAP’s intensive home visiting model uses an accountability tool called Pathways [8, 9]. A Pathway addresses clearly defined actions towards problem resolution and is not considered complete until a measurable outcome is achieved. One participant may be assigned to many different Pathways depending on the problems identified during the initial interview and subsequent home visits [10]. As in most communities, Richland County had geographic areas of health inequality. CHAP used a mapping strategy to determine the census tracts where the unfavorable birth outcomes were disproportionately occurring. The infant mortality rates in Richland County from 2001 to 2005 were 6.7 infant deaths per 1,000 live births for white women, and 17.3 for African-American women [2].

The impact of CHWs has been difficult to document. The Agency for Healthcare Research and Quality (AHRQ) released a report on the outcomes of CHW interventions in 2009, based on 15 different programs, which showed minimal impact on birth outcomes [11]. The CHAP model differs from those programs previously studied in that an accountability measurement tool—Pathways—was used to track each health or social issue a pregnant client faced through to a measurable completion. Additionally, contracts were developed with funders to pay for completed Pathways or outcomes [8, 9].

We evaluated if LBW would be reduced when women at risk of having a LBW infant were provided with intensive home visiting and community based care coordination by CHWs, and Pathways were used to document outcomes. The primary objective was to compare the adjusted odds of LBW between CHAP recipients and non-CHAP recipients. Secondary objectives were a comparison of adequacy of prenatal care and a cost savings evaluation.

## Methods

### The CHAP Intervention

Initially, 4 years of birth certificate data were used to identify where the LBW births were occurring in Richland County. Eligibility for participation in CHAP was based on residence in a census tract with high LBW and poverty rates. Seven census tracts comprised the program-eligible communities; two of these census tracts (6 and 7) represented only six percent of the county population, but almost thirty percent of all county LBW births.

The CHWs that provided home visiting services here were hired from the program-eligible communities and trained at the local community college. CHAP developed an extensive CHW-specific training curriculum that was delivered for college credit. CHWs were supervised by either a registered nurse or physician.

Community health workers (CHWs) functioned as community care coordinators, not providers of direct services, and assisted participants to overcome barriers faced in obtaining necessary health or social services. CHAP developed checklists to be used at each face-to-face home visit encounter between the client and the CHW. A “yes” answer to certain questions triggered the initiation of a defined Pathway. For example, if a client answered “yes” to the question—“Do you need a medical home?”—then a Medical Home Pathway was initiated.

**Fig. 1:**
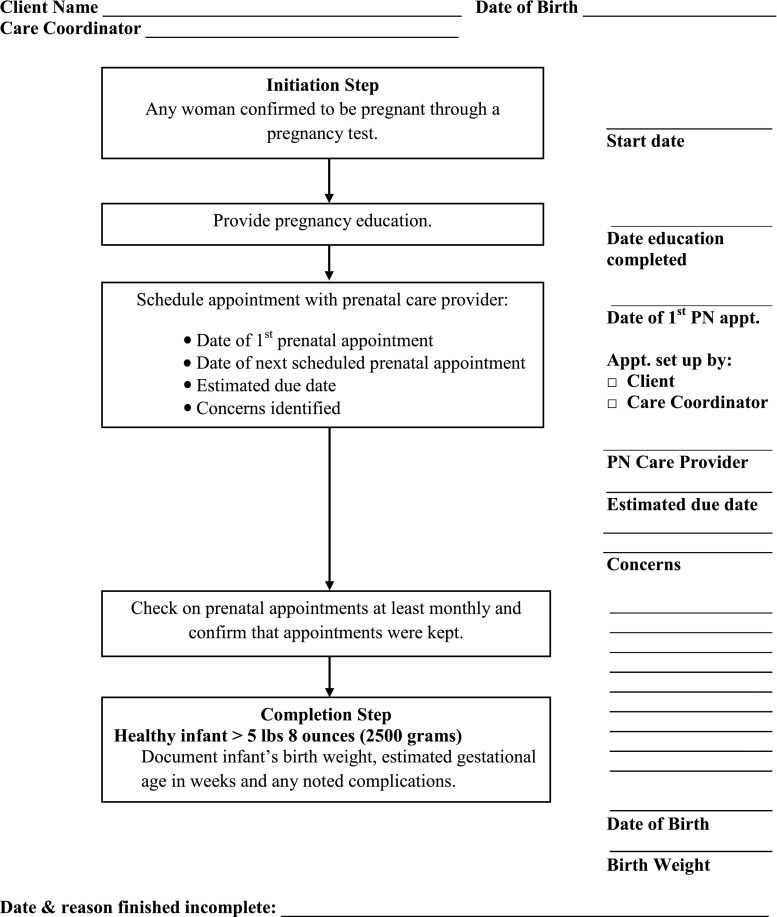
Pregnancy pathway

Pathways are tools to track each identified health or social issue through to a measurable completion or outcome; typically confirmation that the client actually received the medical or social service is required. The Medical Home Pathway tracks the participant’s connection to an ongoing source of primary care and is not documented as complete until the CHW confirms that the client has a medical home. If the client does not connect with a medical home, then the Pathway is closed as “finished incomplete”; recording that the desired outcome was not achieved. In a similar fashion, the Pregnancy Pathway confirms the connection to and maintenance of prenatal care and is not complete until delivery of a viable normal birth weight infant (Fig. 1). A full description of the model can be found in the Agency for Healthcare Research and Quality “Connecting Those at Risk to Care” publications [8, 9].

Contracts were developed between funders and CHAP with payment tied to specific Pathway benchmarks and Pathway completions. In addition, the CHWs received incentive payments if they completed a designated number of Pathways. This strategy improved the accuracy of Pathway tracking within the agency, because monitoring was occurring both programmatically and operationally.

### Study Population and Data Sources

The study was limited to census tracts in which at least five women received CHAP care coordination and gave birth in the time period 2001–2004 (tracts 3, 4, 5, 6, 7, 8, 10 in Richland County, Ohio). Only singleton births were included in the analysis. CHAP medical records were identified for all women meeting the study criteria and all were successfully matched to an Ohio live birth record. Data on the mother’s trimester of enrollment into CHAP and the number of Pathways initiated were extracted from CHAP records. All other study data were from Ohio vital statistics records. Because CHAP clients had more risk factors for LBW than the general population within each census tract, propensity score matching was performed to select a comparison group with a similar distribution of risk factors from Ohio vital statistics records [12, 13] The matching process consisted of estimating propensity scores using a logistic regression model, then matching CHAP clients to controls with similar propensity scores. The logistic regression model was fit to the data from eligible mothers, with CHAP client (yes/no) as the dependent variable. Predictors of CHAP enrollment in this model included mother’s age (<16, 16–18, >18), race (African-American or white), education (if >18 years old: less than high school, high school graduate, one or more years of college), marital status, census tract, and delivery year. All two-way interactions were tested; none were statistically significant and all were dropped from the model. From this logistic regression model, a score reflecting the probability of CHAP enrollment was estimated for each eligible mother.

Next, the propensity score was used in an optimal matching algorithm to match each CHAP recipient to one control. Optimal matching is known to be superior to nearest-neighbor or “greedy” matching [14]. Exact matches for county and delivery year were required.

This study was exempted by the Ohio Department of Health Institutional Review Board and conducted in accord with prevailing ethical principles.

### Analysis

To evaluate the CHAP program’s impact on LBW, logistic regression models were fit to the LBW outcome. First, the unadjusted LBW odds ratio for CHAP mothers versus non-CHAP mothers was calculated. Then, two multivariate logistic regression models were fit, the primary with only non-modifiable risk factors and a secondary also including factors modifiable by the CHAP program. Multivariable adjustment was also appropriate, as propensity score matching and multivariable adjustment are often used in combination to reduce potential bias [15]. The primary model was “non-modifiable only” because it is less likely to over adjust for the mediating effects of CHAP intervention. Covariates included in the primary model were the propensity score matching variables (mother’s age, race, education, marital status, census tract, and delivery year), previous preterm or LBW delivery and tobacco use during pregnancy (none vs. any throughout pregnancy, thus non-modifiable). Other risk factors considered for inclusion in the secondary model were hypertension (chronic or pregnancy-associated), eclampsia, incompetent cervix, renal disease, and uterine bleeding. However, only hypertension was added to the secondary model because there were very few occurrences of the other conditions.

To evaluate the secondary objective, the CHAP program’s impact on the adequacy of prenatal visits, an ordinal logistic regression model was fit to adequate prenatal visits versus less than adequate prenatal visits based on the Kotelchuck index [16]. A logistic regression model was also fit to first trimester prenatal care versus other than first trimester prenatal care.

The number of LBW births prevented was estimated by subtracting the observed number of LBW deliveries from the number expected in the study population if there had been no CHAP intervention. The calculation required the relative risk, for which the odds ratio was considered a sufficient estimate (unadjusted relative risk = 0.43 and unadjusted odds ratio = 0.47). The estimate was taken from the model adjusting for both hypertension (modifiable) and non-modifiable risk-factors. First, the fraction of LBW births not prevented by CHAP was calculated as

$$0.5 + OR {\mkern 1mu} {\mkern 1mu} \times {\mkern 1mu} {\mkern 1mu} 0.5$$

which is the fraction of study women in the non-CHAP group + CHAP risk relative to non-CHAP (CHAP odds ratio) multiplied by the fraction in the CHAP group. Next, the observed number of LBW births was divided by this fraction and rounded to the nearest integer. This method was repeated using the lower and upper confidence limits of the odds ratio to obtain the confidence interval. This method is equivalent to multiplying the preventable fraction (1—odds ratio) by the fraction treated, subtracting that from one and multiplying the reciprocal by the number of observed events [17].

To estimate the potential cost savings of the CHAP program, we first estimated the number of LBW births avoided using the method described above. We then estimated the average cost of delivering the CHAP intervention per client by evaluating the cost per Pathway, cost per client, and the amount paid to CHAP per number of pregnant clients within grant and service contracts. The greatest cost of the program was time spent by a CHW to provide care coordination and the amount of time spent by a CHW was primarily driven by trimester of entry into CHAP.

To evaluate cost savings from LBW births averted by CHAP participation, we applied the average excess LBW costs provided in the 2006 Institute of Medicine (IOM) report [18] to our estimate of LBW births averted. Per IOM, in the first year of life, excess medical expenses per LBW infant are $29,000 and long term costs (including maternal costs, early intervention, special education and lost household and labor market productivity) are $48,275. The dollars saved per dollar invested was calculated by dividing the total cost savings for one prevented LBW infant by the total cost to serve enough pregnant women with Pathways focused care coordination.

## Results

Characteristics of CHAP participants and non-participant controls are summarized in Table 1. The CHAP and non-CHAP groups did not differ significantly (*p* < 0.05) in any of the propensity score variables; the groups are within 2.6 % points for all levels of all propensity score variables with the exception of age, which had a 5.2 % point difference. There were no reported cases of incompetent cervix, uterine bleeding, or renal disease in either group.

**Table 1:** Characteristics of community health access project (CHAP) clients, all non-CHAP mothers* identified from birth certificates, and matched controls

	CHAP clients (n = 115)	Matched controls (n = 115)	All non-CHAP* Births (pre-matching)* (n = 1,443)
*Age*			
<16	16 (13.9 %)	10 (8.7 %)	36 (2.5 %)
16–18	13 (11.3 %)	13 (11.3 %)	122 (8.5 %)
>18	86 (74.8 %)	92 (80.0 %)	1,285 (89.0 %)
*Race*			
African-American	78 (67.8 %)	80 (69.6 %)	325 (22.5 %)
White	37 (32.2 %)	35 (30.4 %)	1,118 (77.5 %)
*Education* ^a^			
Less than HS	28 (32.6 %)	29 (31.5 %)	220 (17.1 %)
High school graduate	36 (41.9 %)	40 (43.5 %)	628 (48.9 %)
Any college	22 (25.6 %)	23 (25.0 %)	436 (34.0 %)
*Marital status*			
Married	17 (14.8 %)	19 (16.5 %)	661 (45.8 %)
Not married	98 (85.2 %)	96 (83.5 %)	782 (52.2 %)
*Census tract*			
3	18 (15.7 %)	20 (17.4 %)	110 (7.6 %)
4	8 (7.0 %)	5 (4.4 %)	188 (13.0 %)
5	20 (17.4 %)	17 (14.8 %)	211 (14.6 %)
6	51 (21.7 %)	26 (22.6 %)	226 (15.7 %)
7	31 (27.0 %)	34 (29.6 %)	159 (11.0 %)
8	5 (4.4 %)	6 (5.2 %)	159 (11.0 %)
10	8 (7.0 %)	7 (6.1 %)	390 (27.0 %)
*Year of birth*			
2001	44 (38.3 %)	44 (38.3 %)	383 (26.5 %)
2002	34 (29.6 %)	34 (29.6 %)	347 (24.1 %)
2003	26 (22.6 %)	26 (22.6 %)	354 (24.5 %)
2004	11 (9.6 %)	11 (9.6 %)	359 (24.9 %)
Tobacco use^b^	45 (39.1 %)	43 (37.4 %)	528 (36.6 %)
Previous preterm or LBW delivery	3 (2.6 %)	2 (1.7 %)	11 (0.8 %)
Hypertension^c^	2 (1.7 %)	4 (3.5 %)	43 (3.0 %)
Eclampsia	2 (1.7 %)	2 (1.7 %)	16 (1.1 %)

* Single birth from census tract 3, 4, 5, 6, 7, 8, or 10

^a^Among mothers >18 years of age

^b^Defined as any tobacco use during pregnancy reported on birth certificate

^c^Chronic or pregnancy-related

A total of 653 Pathways were initiated for the CHAP participants, and all 115 women in this study finished a Pregnancy Pathway (7 were finished incomplete due to LBW). Including the Pregnancy Pathway, CHAP participants had an average of 5.6 Pathways tracked for health and social issues that were identified during the pregnancy and postpartum period. 102 Postpartum and Family Planning Pathways were completed for participants, confirming that 89 % of women attended their postpartum appointments and were using a family planning method. The most common non-medical Pathways initiated were Employment (52 %), Adult Education (50 %), Smoking Cessation (39 %), Food Security (30 %), and Housing (27 %). Two major barriers that were identified to completion of Pathways included transportation and limited community resources for non-medical issues.

Women enrolled in CHAP care coordination from 2001 through 2004 had significantly lower adjusted odds of experiencing a low-birth weight delivery than non-CHAP women [adjusted odds ratio = 0.36, 95 % CI (0.12, 0.96)] (Table 2). There were no significant differences between the adjusted odds of the adequacy of prenatal visits or the timing of the first prenatal visit between CHAP participants and non-CHAP mothers. This finding is different from other home visiting studies that have shown a dosage effect of prenatal home visiting in at-risk women [19, 20].

**Table 2:** Odds ratios and 95 % confidence intervals for pre-term birth

Variable^a^	Unadjusted	Primary model: adjusts for non-modifiable risk-factor covariates^b^	Secondary model: adjusts for all risk-factor covariates^c^
CHAP versus non-CHAP	0.43 (0.16, 1.07)	0.36 (0.12, 0.96)	0.37 (0.12, 1.02)
*Age*			
<16 versus >18		1.58 (0.40, 6.28)	1.17 (0.42, 6.70)
16–18 versus >18		2.13 (0.66, 6.85)	2.11 (0.65, 6.84)
African-American versus White		1.13 (0.35, 3.70)	0.93 (0.28, 3.09)
Not married versus married		3.06 (0.87, 10.0)	4.11 (1.06, 15.92)
Previous preterm or LBW delivery		3.06 (0.50, 18.52)	3.44 (0.55, 21.43)
Tobacco use		4.76 (1.92, 11.84)	5.09 (2.01, 12.87)
Hypertension			6.25 (0.91, 43.16)

^a^Census tract comparisons excluded

^b^Mother’s age (<16, 16–18, >18), race (African-American, white), marital status, census tract, previous preterm or LBW delivery, tobacco use at any time during pregnancy (y/n)

^c^All from primary model and additionally hypertension (chronic and/or pregnancy-associated)

Fifty-six percent of clients in this study entered CHAP in the first trimester of pregnancy, 20 % in the second trimester and 24 % in the third trimester. The estimated cost to provide Pathways community care coordination by CHAP in the time period studied averaged $751 per pregnant client. An estimated 10 LBW births (1 prevented per 11.5 participants) were prevented by participation in the CHAP program from 2001 through 2004 (95 % CI = 1, 17). The cost savings in the first year of life, for each dollar invested in Pathways based community care coordination was $3.36, and the long term cost savings was $5.59 for each dollar invested.

## Discussion

Pregnant women who participated in CHAP, a structured community-based care coordination program provided by CHWs and coupled with Pathways tracking and payment for outcomes, had a significantly lower probability of delivering a LBW infant. CHAP participants living in the targeted census tracts were at an increased risk for poor birth outcomes compared to the general population—67.8 % African-American, 25.2 % age 18 or younger, 85.2 % unmarried, and 39.1 % tobacco users. A challenge to determining the effectiveness of CHW interventions has been identifying a valid control group that effectively accounts for social determinants and their impact on outcomes [21, 22]. Use of an optimal matching algorithm using propensity scores allowed each CHAP recipient to be matched with one control and supported estimation of the number of LBW births prevented.

Areas of health inequalities—whether related to birth outcomes or chronic diseases—can be easily mapped in communities. This study demonstrates the value of identifying communities with disparately poor health outcomes and directly reaching out to individuals within those communities, engaging them through care coordination, connecting them to health and social service interventions, and measuring the results through an accountable measurement tool.

Community health workers perform their work by approaching the whole person—and take into consideration their social, environmental, psychological and health needs in order to impact health outcomes. This is evidenced by the additional Pathways initiated by CHWs in this study for issues related to food security, housing, transportation, employment, and education. These additional Pathways had to be addressed in coordination with preventive health care needs and consideration of the client’s priorities of care. Health and social service siloes exist in communities, and individuals living in poverty often face barriers in accessing these critical services. The community-based care coordinator serves an important role on the healthcare team because of their trusted relationship with the client. They are able to identify key non-medical issues and are skilled in navigating the fragmented health and social service systems.

Some social determinants of health can be addressed at the population level—such as safe drinking water, smoking in public places, elimination of food deserts and safe sidewalks—but individually addressable social determinants also represent a significant intervention opportunity. Housing, education, employment, food security, and many other critical issues can be identified and addressed with effective and accountable care coordination to improve individual progress, reduce stress, and improve health for those individuals at greatest risk.

The CHAP Pathways Model provided the measurement tool to monitor successful connections to both health and social services. Pathways were developed as the pay-for-performance model for CHAP’s contracts and were an important part of the care plan, documentation, and reporting in this study.

There were several limitations in this study. First, although data was collected over a 4-year time period, the total number of women in the CHAP intervention group was small, reflecting the size of program enrollment within the targeted census tracts over the time period studied. A larger sample size would have provided more precise estimates of odds ratios and more power to detect significant differences in all models. Second, there was no random assignment to CHAP intervention or control. Although we attempted to control for bias as much as possible through propensity score matching and covariate adjustment, some selection bias may remain. Additional evaluations, with randomized group assignments, larger numbers of participants, and in different locations are needed to replicate and confirm our findings. Third, the evaluation was limited by the vital statistics records on what cofounders and outcomes we could study. For example, prenatal smoking is potentially modifiable through CHAP with a Pathway that included specific education and support to help patients reduce or quit smoking; however smoking status by trimester was not standard documentation on the Ohio birth certificate. Future work should control for first trimester smoking status and other factors related to low birth weight. Finally, the evaluation was limited by the quality of birth certificate data, which is shown to generally be specific, but not sensitive, as a source of maternal complications [23, 24]. In contrast, birth weight data from the birth certificate has been shown to be more reliable [25].

CHAP may reduce LBW delivery among high risk women through multiple mechanisms. As there were no differences in prenatal care initiation between groups, improvement in early prenatal care does not appear to be one, and this finding is consistent with other studies [26]. However, factors besides medical care are known to impact health outcomes and models of care that address both medical and social factors show promise in reducing LBW [27–30].

This study represents our initial experience with using the Pathways Model to quantify and track care coordination provided to high risk pregnant women. Since the model’s inception, effort has been placed on refining the measurement and tracking process of the Pathways. It was not possible in this study to identify which Pathways specifically led to improved birth outcomes. Newer technology for Pathway tracking has remedied that and can support future research. CHAP participants were initially identified as being at increased risk by where they lived (identified census tracts), but now we have the capability to monitor risk throughout the care coordination period. Our preliminary study can be incorporated into the larger movement to create a national home visiting research network that works to promote the translation of research findings into policy and practice [31].

Starting from an American Academy of Pediatrics—Community Access to Child Health (CATCH) Grant in 2001—the Pathways Model was further developed to embrace multiple care coordination agencies within a service region. The Pathways Community HUB Model is designed to identify the most at-risk individuals in a community, connect them to evidence-based interventions, and measure the results [8, 10]. The HUB Model was developed and piloted by CHAP in 2004 in Richland County, Ohio based on the success of these initial findings. The model was recognized by the Agency for Healthcare Research and Quality (AHRQ) Innovations Exchange and a learning network was established to further study the model [8, 9]. The Pathways Community HUB does not directly provide care coordination services, but subcontracts with care coordination agencies serving vulnerable populations in the community. The community HUB works to coordinate and track progress for all of the agencies within a community providing care coordination. The HUB serves to register and collect focused data on each client served using common Pathways to track quality and outcomes. This model eliminates duplication of care coordination and provides standard quality measurements, allowing care coordination agencies to focus on the most vulnerable community members and strive towards improving overall health outcomes. The Kresge Foundation has recently supported an initiative to develop a standard approach for certification of communities utilizing the Pathways Community HUB Model to assure consistent quality of care coordination.

As stated by CDC, health disparities “must be addressed with intervention strategies related to both health and social programs” [1]. This study shows that structured community-based care coordination coupled with standardized and accountable tracking tools and payment for outcomes may reduce LBW delivery among high-risk pregnant women. The Pathways Model allows for targeting the diversity of needs across racial, ethnic and other sociodemographic distinctions. Identifying communities with disparately poor health outcomes and ensuring the connection of residents to health and social programs can potentially reduce persistent inequalities in health.
